# Recombination rates in pigs differ between breeds, sexes and individuals, and are associated with the *RNF212*, *SYCP2*, *PRDM7, MEI1* and *MSH4* loci

**DOI:** 10.1186/s12711-022-00723-9

**Published:** 2022-05-20

**Authors:** Cathrine Brekke, Peer Berg, Arne B. Gjuvsland, Susan E. Johnston

**Affiliations:** 1 Department of Animal and Aquacultural Sciences, Norwegian University of Life Sciences, Oluf Thesens vei 6, 1433 Ås, Norway; 2 Norsvin, Storhamargata 44, 2317 Hamar, Norway; 3 Institute of Ecology and Evolution, School of Biological Sciences, University of Edinburgh, Charlotte Auerbach Road, Edinburgh, EH9 3FL UK

## Abstract

**Background:**

Recombination is a fundamental part of mammalian meiosis that leads to the exchange of large segments of DNA between homologous chromosomes and is therefore an important driver of genetic diversity in populations. In breeding populations, understanding recombination is of particular interest because it can break up unfavourable linkage phases between alleles and produce novel combinations of alleles that could be exploited in selection. In this study, we used dense single nucleotide polymorphism (SNP) genotype data and pedigree information to analyse individual and sex-specific variation and genetic architecture of recombination rates within and between five commercially selected pig breeds.

**Results:**

In agreement with previous studies, recombination rates were higher in females than in males for all breeds and for all chromosomes, except 1 and 13, for which male rates were slightly higher. Total recombination rate differed between breeds but the pattern of recombination along the chromosomes was well conserved across breeds for the same sex. The autosomal linkage maps spanned a total length of 1731 to 1887 cM for males and of 2231 to 2515 cM for females. Estimates of heritability for individual autosomal crossover count ranged from 0.04 to 0.07 for males and from 0.08 to 0.11 for females. Fourteen genomic regions were found to be associated with individual autosomal crossover count. Of these, four were close to or within candidate genes that have previously been associated with individual recombination rates in pigs and other mammals, namely *RNF212*, *SYCP2* and *MSH4*. Two of the identified regions included the *PRDM7* and *MEI1* genes, which are known to be involved in meiosis but have not been previously associated with variation in individual recombination rates.

**Conclusions:**

This study shows that genetic variation in autosomal recombination rate persists in domesticated species under strong selection, with differences between closely-related breeds and marked differences between the sexes. Our findings support results from other studies, i.e., that individual crossover counts are associated with the *RNF212*, *SYCP2* and *MSH4* genes in pig. In addition, we have found two novel candidate genes associated with the trait, namely *PRDM7* and *MEI1*.

**Supplementary Information:**

The online version contains supplementary material available at 10.1186/s12711-022-00723-9.

## Background

Meiotic recombination is the event in meiosis where double strand breaks are resolved as crossovers, resulting in recombined homologous chromosomes. Thus, recombination leads to variation in haplotypes by breaking up linkage disequilibrium and creating novel combinations of alleles for selection to act upon. In addition, recombination has an important function in the proper segregation of homologous chromosomes during meiosis, and its absence can often lead to nondisjunction and aneuploidy in the resulting gametes [1–4]. Hence, most species have at least one obligate crossover per chromosome pair [5]. However, recombination can also break up beneficial allele combinations that were previously favoured by selection [6] and double strand break formation can increase the risk of mutations and chromosomal rearrangements [7, 8]. These benefits and costs were thought to result in tight regulation of the number of crossovers [9], yet recombination rates have been found to vary to a large degree across a diverse range of taxa [5, 10].

During the last decade, studies of variation in recombination rates have been conducted in a number of mammal populations, including model species such as mice [11, 12], domestic species such as pigs, cattle, and sheep [13–15], and natural populations such as humans, Soay sheep, and red deer [7, 13, 15–17]. Recombination rates often have substantial additive genetic variation in most species studied, with estimates of heritability (${h}^{2}$) ranging from 5% in pigs [15] to 46% in house mice [11]. In addition, most mammals are heterochiasmate (i.e., both sexes recombine, but at different rates), but the direction and degree of the difference between the sexes can vary even between closely related species [18]. Some loci have repeatedly been found to be associated with variation in recombination rates in mammals, including *RNF212*, *RNF212B* and *REC8* [7, 13, 16, 17, 19, 20], which suggests that part of the genetic architecture of this trait is well conserved across species. However, there are also candidate genes that may be specific to the species studied or that for other reasons have not been detected in previous studies [7, 15, 21]. Hence, there is still significant interest in understanding the genetic mechanisms that drive differences in recombination rates between and within populations.

In breeding populations, understanding recombination is of particular interest because it can break up undesired linkage phases and produce novel combinations of alleles that could be exploited in selection. A higher recombination rate may help quantitative traits to respond to selection faster [22], as it can increase the additive genetic variance available for selection [23]. This potential advantage has led to the long-standing theory that domestication has indirectly selected for increased recombination rates in domestic mammals [18], although this view has been challenged by more recent studies that found that domesticated species have similar or lower recombination rates than their wild counterparts [24].

In this study, we used genotype data at more than 50,000 single nucleotide polymorphisms (SNPs) and extensive pedigrees for more than 250,000 pigs from five domestic pig breeds (*Sus scrofa*) to study the genetic architecture and variation in individual autosomal crossover count (ACC). Our objectives were to: (a) construct high-density linkage maps to characterize sex-specific recombination landscapes; (b) characterise the genetic architecture of ACC by estimating its heritability and identifying genomic regions associated with its genetic variation; and (c) examine differences in ACC and its genetic architecture between breeds and the sexes.

## Methods

### Breeds

This study focused on five purebred commercial breeding populations with pedigree and genotype data: two sow breeds, Landrace (LR) and Large White (LW); and three boar breeds, Duroc (DU), Synthetic (SY) and Pietrain (PI).

### Genotype data

Genotypes were available from two medium-density SNP chips: an Illumina GeneSeek custom 80K SNP chip and an Illumina GeneSeek custom 50K SNP chip, which had 50,705 SNPs in common. The physical positions of the SNPs were determined based on the Sscrofa11.1 reference genome. The genotype data were filtered for each breed separately to remove SNPs with minor allele frequencies lower than 0.01, a genotype call rate lower than 0.95, and/or those that showed strong deviations from the Hardy Weinberg equilibrium (χ^2^ > 600). The sex chromosomes were not included in the study. An overview of the number of SNPs and animals from the five populations is in Table 1. This dataset is referred to as the 50K set and was used for determining individual autosomal crossover counts (ACC). A set of imputed genotypes to 660K (Axiom Porcine Genotyping Array) was available for all breeds and individuals. This dataset is referred to as the 660K set and was used for the genome wide association analysis.

**Table 1:** Overview of the 50K genotype data

Breed	Numbers of			
	SNPs	Animals	Males	Females
Landrace (LR)	50,705	70,943	11,685	59,258
Duroc (DU)	50,705	17,137	8397	8740
Large White (LW)	50,705	95,613	32,683	62,930
Pietrain (PI)	50,705	22,784	15,009	7775
Synthetic (SY)	50,705	50,818	30,198	20,620

Number of animals and SNPs in the datasets after filtering for a minor allele frequency lower than 0.01, a call rate lower than 0.95, and a strong deviation from Hardy–Weinberg equilibrium (χ^2^ > 600). SNPs on sex chromosomes and unmapped SNPs were also removed

### Full-sib family pedigrees

**Fig. 1:**
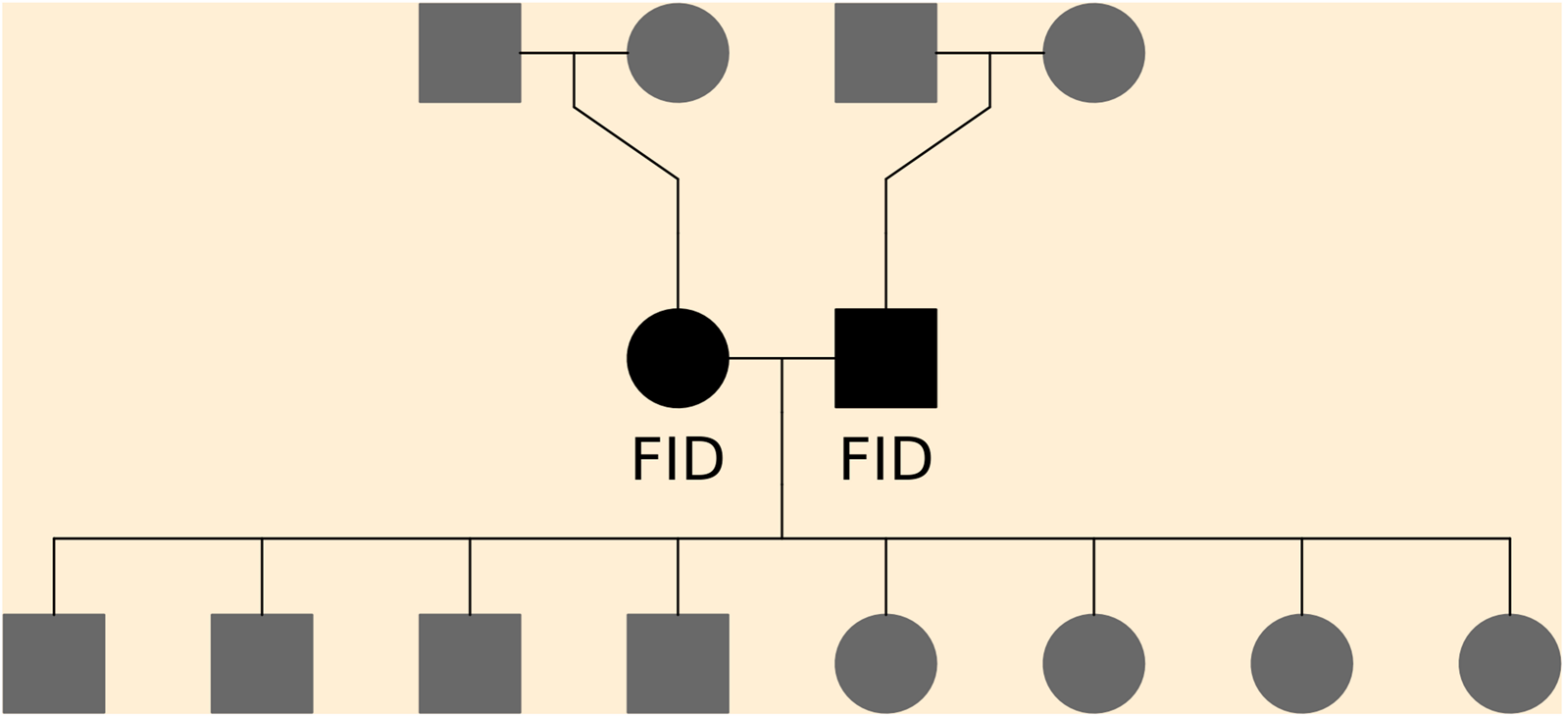
Illustration of the full sib family structures. The focal individuals (FID) are the parents, in black, and the crossover events studied are those in the gametes transmitted from the FID to the offspring

For each breed, we sub-divided pedigrees into three-generation full-sib families that each included a unique dam and sire mating pair combination (hereafter referred to as focal individuals, or FID), along with their parents and offspring. This family structure allows for phasing of the FID and offspring genomes and determining the positions of crossovers that occurred in the gamete transmitted from a FID to its offspring. A FID can be in multiple full-sib families (i.e., when mating with a different individual), but each individual gamete from a FID is only counted once. An example of a three-generation full-sib family is illustrated in Fig. 1. Only families that included genotypes on at least one offspring, on both FID (parents), and on all four grandparents were included. An overview of the numbers of families and unique FID is in Table 2.

**Table 2:** Overview of the full-sib family datasets

Breed	Numbers of				
	Families	Gametes	Offspring/family	Sires	Dams
Landrace (LR)	7295	74,534	1–24	319	4808
Duroc (DU)	5101	18,365	1–18	192	1687
Large White (LW)	6845	82,196	1–24	273	4695
Pietrain (PI)	2370	24,198	1–27	196	1353
Synthetic (SY)	4437	51,245	1–20	224	2633

Sires and dams are the number of unique male and female FID in each breed

### Linkage mapping

The markers were physically mapped to the Sscrofa11.1 reference genome by extracting flanking sequences for each marker from the chip manifest files and aligning them to the 11.1 reference genome using the bwa software [25]. Breed-specific linkage maps were constructed using the LepMap3 software [26] with the linkage groups and marker ordering from the physical mapping. The *filtering2* module was run to filter markers based on segregation distortion, with *dataTolerance* = 0.01, as recommended by Lepmap3 for multi-family datasets. The *Separatechromosomes2* module of the LepMap3 software was used as a filtering step (rather than to identify the linkage groups from scratch) by running the SNPs from a given chromosome based on the physical map through the module and excluding SNPs that were not assigned (LOD score < 5) to the largest linkage group. The number of SNPs in the final linkage map is provided in Table 3. Then, the *Ordermarkers2* module of LepMap3 was used to calculate the centimorgan (cM) positions for the SNPs using the Haldane mapping function. The ordering of the SNPs was based on their physical positions on the reference genome. Recombination rates from linkage map data were defined in cM per megabase (Mb) [10].

**Table 3:** Estimates of linkage map lengths by line, sex, and chromosome

Chr	Mb	N_SNPs_	Linkage map length (cM)									
			LR		DU		LW		PI		SY	
			M	F	M	F	M	F	M	F	M	F
1	274.3	4858	145.2	122.9	156.8	128.1	158.9	142.0	145.8	129.9	159.3	134.8
2	151.9	3306	109.5	129.9	108.8	131.3	117.8	149.0	107.9	129.5	115.8	138.5
3	132.9	2912	111.6	133.5	112.2	131.5	121.0	154.7	113.4	130.4	120.4	140.2
4	130.9	3002	104.2	132.0	102.6	133.4	110.2	151.5	103.3	135.9	108.7	140.4
5	104.5	2290	98.1	140.8	98.4	136.0	103.6	156.8	96.8	144.2	105.1	148.5
6	170.8	3440	119.6	149.3	125.9	156.8	130.3	179.4	120.8	149.8	127.7	162.5
7	121.8	2751	113.4	137.2	112.3	137.8	117.6	158.6	107.8	140.0	115.6	146.6
8	139.0	2924	102.1	122.2	103.1	124.3	105.4	137.6	104.0	126.8	106.3	132.6
9	139.5	3168	104.7	142.0	103.6	143.5	107.3	163.7	104.7	147.8	108.8	156.5
10	69.4	1510	93.9	121.3	91.8	124.3	98.0	138.3	88.3	131.3	94.7	131.4
11	79.2	1846	69.7	114.4	67.8	110.0	79.3	122.6	63.9	120.6	78.3	117.8
12	61.6	1296	78.3	122.0	77.3	118.0	86.5	133.7	71.3	123.6	82.4	127.2
13	208.3	3669	116.9	109.5	126.0	116.9	127.5	123.4	117.6	115.5	126.1	117.6
14	141.8	3284	105.3	124.7	109.8	126.8	114.8	151.9	105.8	124.4	112.7	137.0
15	140.7	2916	98.9	113.0	101.7	115.6	104.9	124.8	99.8	116.4	106.0	119.8
16	79.9	1829	67.0	105.5	69.9	103.6	77.0	111.3	63.2	110.5	74.4	108.4
17	63.5	1399	61.4	106.0	71.9	106.3	69.6	115.7	60.5	106.4	69.3	113.2
18	56.0	1257	54.5	89.5	54.6	87.4	57.6	100.3	56.1	94.7	59.4	95.6
Total	2266.1	47,657	1754.4	2215.7	1794.4	2231.3	1887.1	2515.4	1731.1	2277.8	1871.2	2368.7

*Chr* chromosome, *Mb* physical length in megabases, *N*_*SNPS*_ number of SNPs in each linkage map after filtering in LepMap3, *Total* total autosomal linkage map length in cM, *M* is for male and *F* is for female, *LR* Landrace, *DU* Duroc, *LW* Large White, *PI* Pietrain, *SY*: Synthetic

### Individual autosomal crossover counts

Individual recombination rates were measured as the autosomal crossover count (ACC). Crossovers were counted from the gamete phase from the output of the *orderMarkers2* module of LepMap3 for offspring, and assigned to the appropriate parent, i.e., the FID in which the meiosis took place.

### Additive genetic variation for autosomal crossover counts

We estimated variance components for individual ACC with the following repeatability model using the average information (AI) algorithm for the restricted maximum likelihood (REML) method in the DMU v6 software [27]:

$${\mathrm{Y}}_{ij}={\mathrm{sex}}_{i}+ {\mathrm{b}}_{1}*{\mathrm{age}}_{i}+{\mathrm{id}1}_{i}+{\mathrm{id}2}_{i}+{\mathrm{b}}_{2}*{\mathrm{het}}_{i}+{\mathrm{e}}_{ij},$$

$\mathrm{Y}$ is the ACC in gamete (observation) $j$ transmitted from FID $i$, $\mathrm{sex}$ is the fixed effect of the sex of the FID, ${\mathrm{b}}_{1}$ is the fixed regression of age of the FID when the offspring is born (from ages 1 to 4), $\mathrm{id}1$ is the random additive genetic effect of the FID, $\mathrm{id}2$ is the random effect of the permanent environment of the FID (i.e. environmental effects that are constant across repeated measures on an FID), $\mathrm{het}$ is the method-of-moments F coefficient estimates of the FID $i$ calculated with the *–het* function in PLINK1.9 [28], ${\mathrm{b}}_{2}$ is the regression of ACC on $\mathrm{het}$ of the FID $i$, and ${\mathrm{e}}_{ij}$ is the residual effect of observation $j$ from FID $i$. The narrow-sense heritability (${h}^{2}$) was defined as the proportion of phenotypic variance explained by the additive genetic effect and was estimated separately for each breed and sex.

### Genome-wide association study

Genome-wide associations were conducted using the 600K datasets with the *fastGWA* module implemented in GCTA [29], using the mean ACC per FID as the response variable. This module uses a mixed model with a sparse genomic relatedness matrix (GRM) to correct for relatedness and principal components to control for population stratification. The sparse GRM was calculated with the *–make-bK-sparse* option of GCTA using a cut-off value of 0.05 based on the full genomic dataset, i.e., off-diagonal elements below 0.05 were set to 0 [29]. The proportion of variance explained by a SNP associated with the trait ($\mathrm{PVE}$) was computed as:

$$\mathrm{PVE}=\frac{2{\widehat{\upbeta }}^{2}\mathrm{MAF}(1-\mathrm{MAF})}{2{\widehat{\upbeta }}^{2}\mathrm{MAF}\left(1-\mathrm{MAF}\right)+{\left(\mathrm{SE}\left(\widehat{\upbeta }\right)\right)}^{2}2\mathrm{NMAF}(1-\mathrm{MAF})},$$

$\mathrm{MAF}$ is the minor allele frequency for the SNP, $\widehat{\upbeta }$ is the estimated effect size (i.e. the slope) of the allele, SE($\widehat{\upbeta }$) is the standard error of the estimated effect size and $\mathrm{N}$ is the sample size, i.e. the number of individuals in the analysis.

All analyses and data manipulation where no other software is mentioned were carried out in R 3.6.3 [30].

## Results

### Linkage maps

**Fig. 2:**
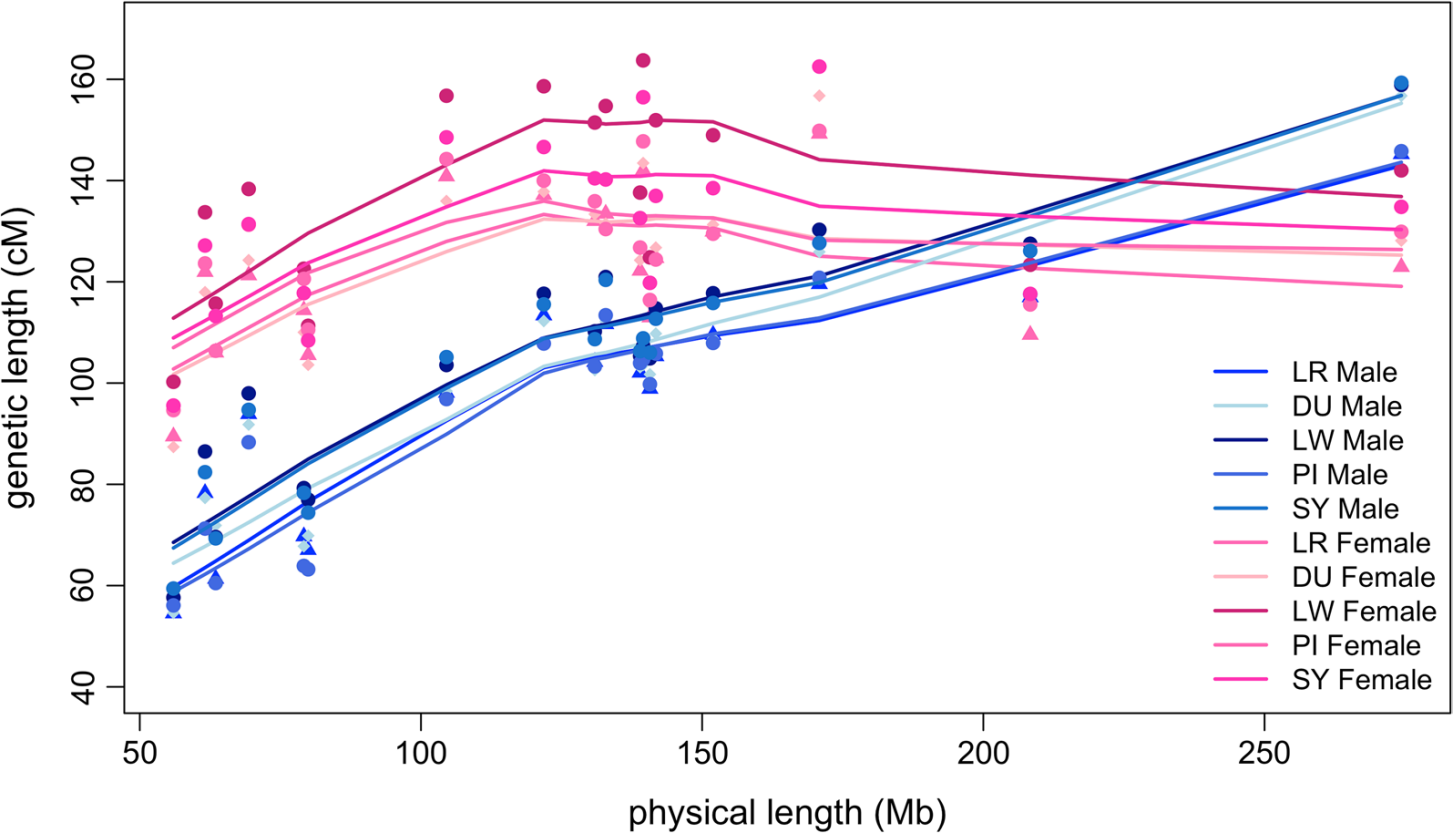
Relationship between the physical (Mb) and genetic (cM) length of the chromosomes. The relationship between the genetic length in cM (x-axis) and physical length in Mb (y-axis) is plotted for each chromosome by breed and sex. The relationship is plotted with robust locally weighted regression using the Lowess smoother in R

**Fig. 3:**
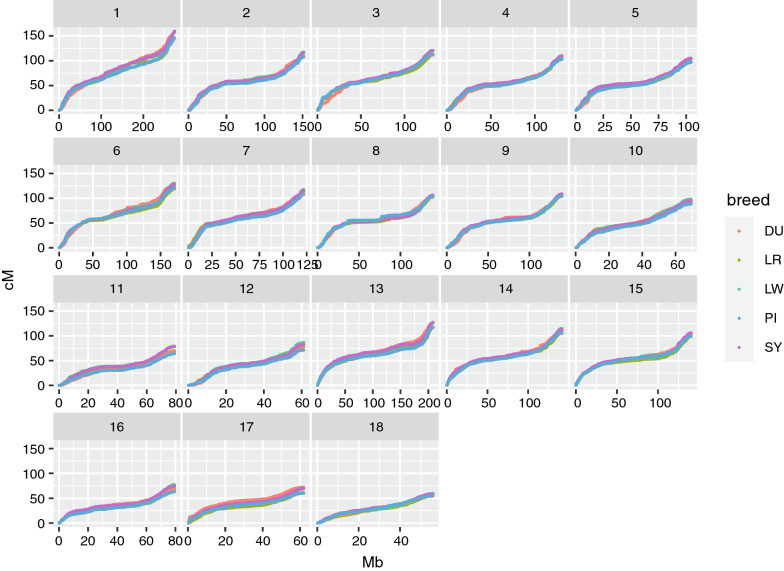
Male autosomal linkage maps by breed. Genetic positions of the SNPs in cM are plotted against their physical positions in Mb. Chromosome numbers are given in the facet headers

**Fig. 4:**
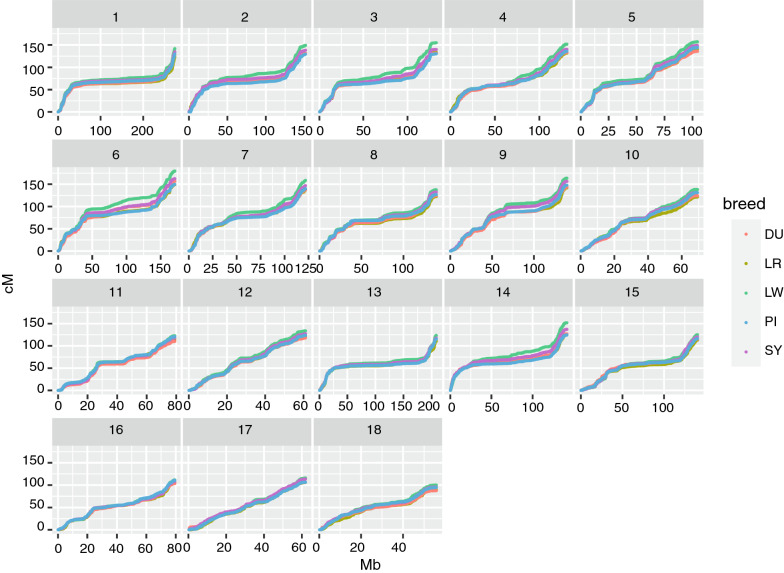
Female autosomal linkage maps by breed. Genetic positions of the SNPs in cM are plotted against their physical positions in Mb. Chromosome numbers are given in the facet headers

**Fig. 5:**
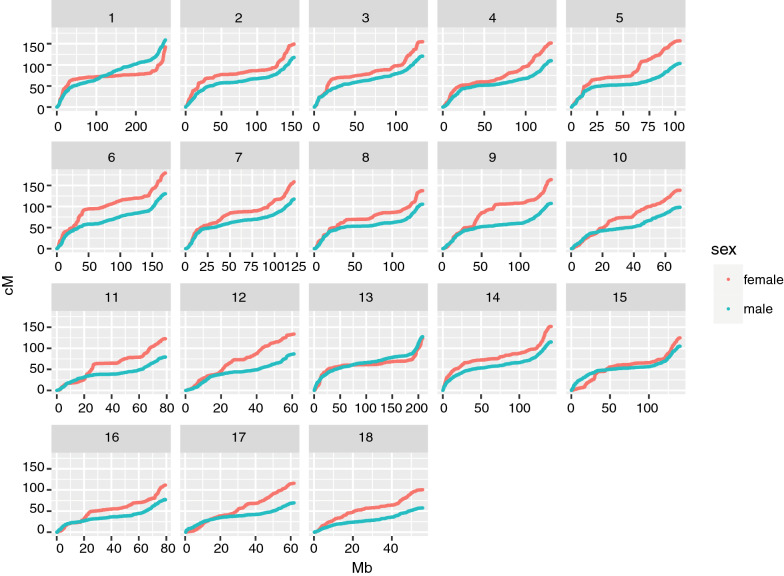
Comparison of male linkage maps (blue) and female linkage maps (red) of the large white breed. Genetic positions of the SNPs in cM are plotted against their physical positions in Mb. Chromosome numbers are given in the facet headers

The sex-specific autosomal linkage maps spanned a total length of 1731.1 cM (PI breed) to 1887.1 cM (LW breed) for males and of 2231.3 cM (DU breed) to 2515.4 cM (LW breed) for females (Table 3). The LW breed had the highest recombination rate (cM/Mb) among the five breeds for both sexes. The linkage map lengths of each chromosome for each breed are in Table 3. For all breeds, recombination rates were higher for females than males for all chromosomes, except chromosomes 1 and 13, for which the male rates were slightly higher. The relationship between physical length (Mb) and map length (cM) of the chromosomes was close to linear for males, but clearly non-linear for females, as shown in Fig. 2 based on robust locally weighted regression [31]. Within chromosomes, the recombination rates were elevated towards the telomeres in both sexes for all chromosomes, including the acrocentric chromosomes 13 to 18. The total recombination rate and pattern along the chromosomes were more similar for the same sex between breeds (Figs. 3 and 4) than between the sexes from the same breed (see for example, the comparison of male and female maps for the LW breed, Fig. 5).

## Genetic variation in individual autosomal crossover counts

**Fig. 6:**
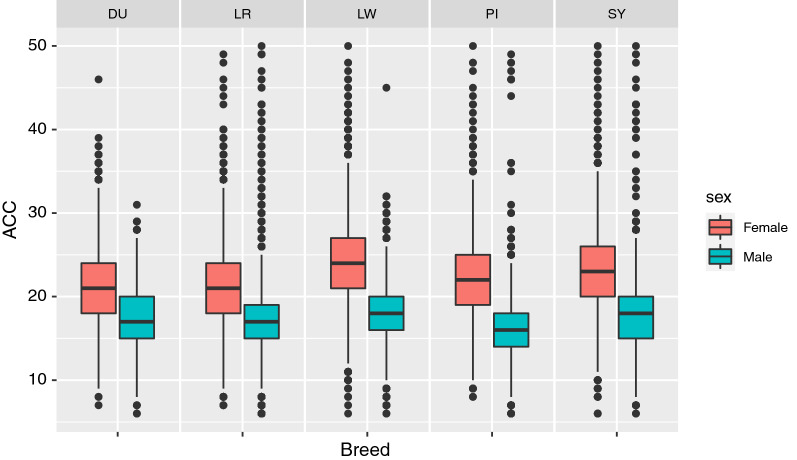
Distribution of the autosomal crossover count by breed and sex. The blue boxes are counts for males and red boxes are counts for females for each breed. The midline is the median and the box is from the 25th percentile to the 75th percentile

The average ACC per gamete ranged from 16.3 (PI) to 18.2 (LW) in males and from 21.3 (LR) to 24.4 (LW) in females. The ACC was close to normally distributed (see Additional file 2: Figure S1), with a greater standard deviation in females than in males (Fig. 6). Observations (gametes) with less than six ACC or more than 50 ACC were excluded.

Results from the genetic analysis of ACC are in Table 4. Heritability estimates for ACC ranged from 0.04 (SE = 0.01) (SY) to 0.07 (SE = 0.02) (DU) in males and from 0.08 (SE = 0.01) (DU and PI) to 0.11 (SE = 0.01) (LR and LW) in females. For all breeds, heritability estimates were higher in females than in males and the phenotypic variance of ACC was substantially larger in females than in males for all breeds, except LR. Most of the phenotypic variance was explained by the error term for all breeds and both sexes. A higher inbreeding coefficient of the FID was associated with reduced ACC (see Additional file 1: Table S1).

**Table 4:** Basic statistics and variance component estimates for ACC by breed and sex

Breed	Sex	N_FID_	N_obs_	Mean (SD)	${h}^{2}$ (SE)	V_p_	V_e_
LR	Female	4808	37,148	21.3 (4.2)	0.11 (0.01)	18.53	16.40
LR	Male	319	37,386	16.8 (3.5)	0.05 (0.05)	20.34	10.92
LW	Female	4695	41,092	24.4 (4.6)	0.11 (0.01)	21.41	19.08
LW	Male	273	41,104	18.2 (3.4)	0.06 (0.01)	11.44	10.81
DU	Female	1687	9268	21.4 (4.3)	0.08 (0.01)	18.35	16.55
DU	Male	192	9097	17.4 (3.3)	0.07 (0.02)	10.69	9.89
SY	Female	2633	25,623	22.7 (4.4)	0.10 (0.01)	19.73	17.45
SY	Male	224	25,622	17.6 (3.4)	0.04 (0.01)	11.38	10.83
PI	Female	1353	12,101	21.8 (4.4)	0.08 (0.01)	19.08	16.89
PI	Male	196	12,097	16.3 (3.3)	0.06 (0.02)	10.89	10.19

*N*_*FID*_ total number of FID (with repeated measures), *N*_*obs*_ total number of observations (meioses) in each sex and line, *Mean* mean ACC with standard deviations in parenthesis, ${h}^{2}$ heritability estimate with standard errors in parenthesis, V_p_ and V_e_ phenotypic variance and error variance, respectively, *LR* Landrace, *DU* Duroc, *LW* Large White, *PI* Pietrain, *SY* Synthetic

### Genome-wide association study

**Fig. 7:**
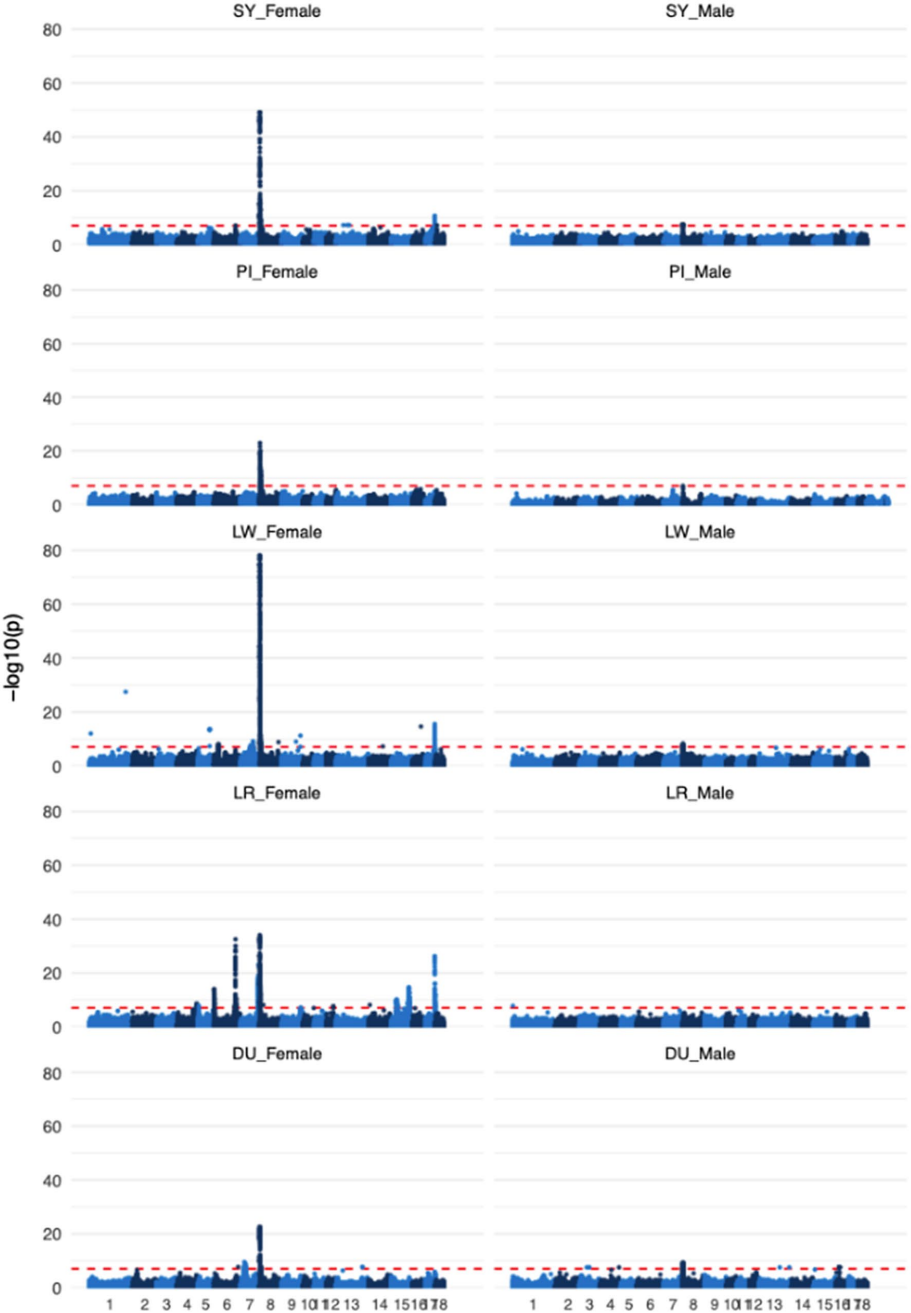
Genome-wide associations between SNPs and autosomal crossover count. Genome-wide associations between SNPs and autosomal crossover count for each breed and sex. The dotted line is the statistical significance threshold = 0.05/number of markers per analysis. The Y axis is the negative logarithm of the p-value and the x axis is the physical positions of SNPs with alternating colors from autosome 1 to 18

We found 14 genomic regions that were significantly associated with mean ACC in females and one genomic region in males (Table 5). Base pair positions and results from the GWA analysis for the top SNPs for all peaks and breeds are in Table 5 and the results for all breeds and both sexes are plotted in Fig. 7. A region on chromosome 8 was significant for both males and females and showed the strongest association for both sexes; this region was significantly associated with ACC of females for all breeds and with ACC of males for four of the five breeds, with p-values for the top SNPs ranging from 2.9 × 10^–23^ to 7.4 × 10^–79^ in females and from 2.9 × 10^–8^ to 5.9 × 10^–10^ in males. The top SNPs were between 0.15 (LR female) and 1.13 (PI female) Mb away from the *RNF212* gene, which is known to be associated with individual recombination rates in several other mammals, including humans [19], cattle [21], Soay sheep [17], and pigs [15]. Chromosome 17 had a significant region for females in breeds LR, LW, and SY, where the top SNPs in LR and SY were within the *SYCP2* (*synaptonemal complex protein 2*) gene, while the top SNP for LW females was 0.03 Mb away from the *SYCP2* gene. Chromosome 6 had two significant regions for LR females. For the first region, the most significant SNP (P = 1.36 × 10^–14^) was 2 kb from the *PRDM7* (*PR domain-containing protein 7*) gene. The second region on chromosome 6 was also significant in the SY females, with the top SNPs 0.03 (LR) and 0.29 (SY) Mb removed from the *MSH4* (*MutS homolog 4*) gene. LW females also had a significant region on chromosome 6, but with no clear candidate gene in close proximity. Chromosome 5 had a significant region for LR females, with the top SNP (P = 1.54 × 10^–8^) located within the *MEI1* (*meiotic double-stranded break formation protein 1*) gene. The LR, LW, and DU breeds had a significant region on chromosome 7 for females but with no candidate genes in immediate proximity to any of the top SNPs. However, it should be noted that chromosome 7 includes the *REC8* and *RNF212B* genes, which have been associated with individual recombination rates in several other mammal species [13, 16, 20, 32], but these genes are relatively far away from the top SNPs (see Table 5). Analysis of LR females showed significant associations for five additional regions; one on chromosomes 4, 9, and 12, and two on chromosome 15. However, we did not identify any likely candidate genes involved in meiosis or recombination near any of these five peaks.

**Table 5:** Top SNPs identified in genome-wide association analyses of ACC for each breed and both sexes

Chr	Breed	Sex	SNP position	Frequency of allele 1	Effect of allele 1	SE	P-value	% V_p_	Candidate gene
4	LR	F	124741468	0.20	0.41	0.07	2.70E−09	0.73	
5	LR	F	6863929	0.45	0.32	0.06	1.54E−08	0.66	*MEI1*
6	LR	F	61205	0.18	0.55	0.07	1.36E−14	1.22	*PRDM7*
	LW	F	28051609	0.20	0.48	0.08	7.14E−09	0.71	
	LR	F	137566317	0.18	0.86	0.07	3.02E−33	2.91	*MSH4*
	SY	F	137825063	0.48	0.42	0.08	8.51E−08	1.08	
7	LW	F	78531203	0.39	0.43	0.07	6.83E−10	0.80	*RNF212B*/REC8*
	LR	F	113235081	0.41	0.56	0.06	9.39E−24	2.06	
	DU	F	23426839	0.34	− 0.72	0.12	3.19E−10	2.29	
8	LR	F	562791	0.06	1.47	0.12	8.51E−35	3.06	*RNF212*
	LW	F	164462	0.29	− 1.37	0.07	7.35E−79	7.00	
	LW	M	1197996	0.36	− 0.72	0.12	4.76E−09	12.28	
	SY	F	164462	0.63	1.16	0.08	7.16E−50	7.73	
	SY	M	144871	0.65	0.60	0.11	2.89E−08	12.79	
	DU	F	178951	0.18	− 1.41	0.14	2.87E−23	5.53	
	DU	M	794886	0.20	− 1.03	0.17	5.94E−10	16.87	
	PI	F	1543451	0.18	1.34	0.13	9.50E−24	6.95	
	PI	M	1324636	0.24	0.87	0.15	3.11E−09	15.39	
9	LR	F	123194749	0.43	0.30	0.06	6.95E−08	0.60	–
12	LR	F	43697634	0.39	0.32	0.06	2.35E−08	0.65	–
15	LR	F	115153652	0.20	− 0.55	0.07	2.03E−15	1.30	–
	LR	F	35743431	0.35	− 0.37	0.06	1.02E−10	0.86	–
17	LR	F	59924616	0.27	− 0.67	0.06	6.05E−27	2.35	*SYCP2*
	LW	F	59845939	0.50	− 0.55	0.07	2.58E−16	1.41	
	SY	F	59911489	0.36	− 0.53	0.08	2.32E−11	1.67	

*SNP* positions are in base pairs, *SE* standard error of the effect, *P* P value, %V_p_ is the proportion of phenotypic variance explained by the SNP, *LR* Landrace, *DU* Duroc, *LW* Large White, *PI* Pietrain, *SY* Synthetic

^*^Relatively distant from the peaks; *REC8* position: 75118744–75139630 and *RNF212B* position: 75796882–75829620

## Discussion

In this study, we confirm previous findings by Tortereau et al. [33] and Johnsson et al. [15] that recombination rates in the domestic pig vary between the sexes, along the genome, between and within breeds, and that there is a heritable component to the variation between individuals. Sex explained most of the variation in genome-wide rates and distribution of recombination. We found that a genomic region near the *RNF212* gene strongly influences individual crossover rates for all breeds and for both sexes. We also identified ~ 13 other genomic regions that are associated with individual crossover rates in one or some of the breeds, or only for one sex. Some of these genomic regions were in or near genes with known functions in meiosis, including *SYCP2*, *PRMD7* and *MSH4*. In the following sections, we discuss these results in greater detail, provide context to how they may aid in better understanding the genetic mechanisms behind recombination rate, and their implications for commercial breeding populations.

### Genome-wide recombination rates differ between breeds, but recombination landscapes do not

The average genetic map lengths (in cM) estimated in our study are more in line with those reported by Tortereau et al.[33] than with those reported by Johnsson et al. [15], who found slightly higher estimates, but also reported potential overestimation of total genetic map lengths. However, the pattern of recombination rate along the chromosomes is well conserved across breeds (Figs. 3 and 4). This similarity in patterns could be an artefact due to differences in marker densities or due to runs of homozygosity in some regions of the genome, which make it difficult to detect recombination events. However, there was a substantial difference in recombination patterns between the sexes that cannot be explained by these artifacts, assuming that there are no differences in marker densities or runs of homozygosity between the sexes. This suggests that the observed patterns are highly likely to reflect the variation in recombination rates along the genome. The genome-wide rate of recombination also differed between breeds, in agreement with previous studies [15, 33, 34]. These differences were mainly driven by the largest chromosomes, for which it is more common to have more than the obligate crossover due to reduced effects of crossover interference [35]. Breed differences were larger for females than for males. Results also showed that genome-wide crossover counts were affected by inbreeding, where FID with higher inbreeding coefficients tended to have lower crossover counts (see Additional file 1: Table S1). The most likely reason for this observation is that higher levels of recent inbreeding can lead to longer runs of homozygosity, which may limit the ability to detect double crossovers that occur within these regions [17].

### Recombination landscapes and rates differ between the sexes

In this study, we observed a substantial difference in recombination rates between sexes for all five breeds, with females showing ~ 1.28 times more crossovers than males. This direction of heterochiasmy is common in mammalian recombination, and while there has been great interest in determining the mechanisms that underlie this sexual dimorphism, there are few compelling explanations for it [36], since the direction and degree can differ even between closely-related species. In the literature, there is some evidence for a molecular basis to heterochiasmy. For example, in humans, there is evidence for selection against non-recombinant chromatids in meiosis II [37]. In addition, there can be sex differences in the packing of the chromosomes in the early prophase of meiosis, with differences in synaptonemal complex length between the sexes that correlate with recombination rates in humans [38], bovid species [39], and even *Arabidopsis* [40]. There may also be evolutionary drivers for heterochiasmy, such as differences in haploid selection between the sexes [41], sexual dimorphism and sperm competition [42], and the effects of meiotic drive [36], but empirical evidence for these mechanisms remains limited. Nevertheless, some of these mechanisms may explain the sex differences in the domestic pig, particularly mechanisms related to the synaptonemal complex. However, some sex differences observed in our study may be driven by differences in the number of male and female FID due to the breeding structure of the pigs, i.e. each male FID had on average ~ 150 offspring in the dataset, while each female FID had only ~ 7. However, the total number of meiosis events investigated is the same for males and females and differences in rates between sexes are also found in studies where the numbers of male and female FID are almost the same, e.g. in humans [43].

### Genetic variation in individual crossover count

The heritability estimates of individual crossover counts were low but significantly different from zero, in agreement with Johnsson et al. [15] and Lozada-Soto et al. [42]. Estimates of both heritability and phenotypic variance were lower for males than for females, which is consistent with what has been found for many other mammalian species [15–17, 45]. In general, the standard errors of the heritability estimates were higher for males than for females, which may be due to the smaller number of unique male FID and the lower heritability estimates, leading to more uncertainty in the estimates (Table 4). Most of the phenotypic variance was explained by the error terms (Table 4), possibly because each crossover that occurs during meiotic division has a 50:50 chance of segregating into a particular gamete. This Mendelian sampling of crossovers can lead to variation in the number of crossovers inherited in the gametes characterised in this study. Another factor which may affect estimates of phenotypic variance and heritability is that we can only measure recombination in gametes that result in live animals, which excludes the other products of meiosis (i.e. polar bodies and/or eggs in females and sperm cells in males that do not end up in a live offspring). If there is selection on crossover count, then the analysis is biased towards a sample of successful gametes, meaning that the true phenotypic variance may be underestimated, which may in turn increase heritability estimates. Indeed, a study of recombination in all products of female meiosis in humans showed selection against non-recombinant chromatids in meiosis II [37]. In the future, it would be of great interest to study more of the products from meiosis in these pig breeds to identify potential signs of selection between gametes.

### Genome-wide association analysis of autosomal crossover count

The strongest association with individual ACC is in a region on chromosome 8, close to the *RNF212* gene, which has been found to be associated with individual recombination rates in several mammalian species, including other pig breeds [15, 16, 19–21, 46]. This region is the only one that showed an association with individual ACC in all five breeds. A study by Reynolds et al. [47] in mice established that the *RNF212* gene has a dosage sensitive effect on crossover rates and that *RNF212*-knockout mice are sterile, implying a critical role in chromosome segregation and fertility. For the most highly associated SNP close to the *RNF212* gene, in the LR and PI breeds the frequency of the allele associated with increased ACC was low (Table 5), and these same two breeds also had the lowest mean ACC (Table 4.) However, the higher mean ACC for the LW breed did not appear to be explained by the allele frequencies in this region. It should also be noted that none of the top SNPs in this region for the five breeds occur directly within the *RNF212* gene, suggesting that the causal variant may be regulatory rather than protein-coding, or that the linkage phase between the top SNP and the causal variant may differ between the breeds.

Females from the LR and SY breeds had a highly associated SNP within the *SYCP2* gene on chromosome 17 and LW females a top SNP at 0.03 Mb from this gene. The *SYCP2* gene encodes a protein that is part of the axial elements along which the chromatids are organized in the early prophase of meiosis, which together with SYCP2 proteins, become the lateral elements of the synaptonemal complex during synapsis [48]. The effect of the *SYCP2* gene on ACC may be related to the correlation between synaptonemal complex length and genetic length discussed earlier. In humans, Halldorsson et al. [7] found an association of ACC with the *SYCE1* gene, which encodes one of the other synaptonemal complex proteins, and another study in pigs by Johnsson et al. [15] also found an association with individual recombination rates in close proximity to the most highly associated SNPs on chromosome 17 identified here.

Three genomic regions associated with ACC were identified on chromosome 6. One region was identified for the LR females and may be associated with a gene that is annotated as *PRDM7* in the NCBI annotation release 106, although it was identified as *PRDM9* by Johnsson et al. [15]. *PRDM7* is a gene with a high level of homology to *PRDM9* [49], a gene that specifies the positioning of recombination hotspot in mammals, with different alleles directing double-strand break and crossover formation to particular DNA sequence motifs [50]. Whilst *PRDM9* is primarily viewed as affecting crossover positioning rather than crossover rates, it has been associated with genome-wide crossover rates in cattle [14]. This may be due to differences in the abundance of DNA sequence motifs corresponding to different *PRDM9* alleles that occur in cattle, and we hypothesise that a similar mechanism may underpin the association observed with *PRDM7* in the current study. A second region on chromosome 6 was identified for the LR and SY females and contained the *MSH4* gene, which encodes a meiosis-specific protein essential for reciprocal recombination [51] and has been associated with individual recombination rates in humans [7] and pigs [15]. Finally, a region on chromosome 5 was associated with ACC in LR females and contained the *MEI1* gene, which encodes a protein that is involved in double-strand break formation during meiosis [52], but to our knowledge has not been previously reported to be associated with individual recombination rates.

Several of the genomic regions reported here were only identified for one or two breeds or only for females. This may imply sexual dimorphism in the genetic architecture of ACC in pigs but it may also be explained by the difference in the number of focal individuals for males versus females. The differences in identified associations between breeds may also be due to differences in allele frequencies and/or linkage disequilibrium; the LR breed has been a closed breeding line since 1958 and may very well differ from the other breeds in some of these regions either due to drift or as a consequence of selection, which may explain the identified associated genomic regions that were unique to LR. Lozada-Soto et al. [44] also conducted a genome-wide association analysis of individual recombination rates in Large White and Landrace pig breeding lines, but none of the regions that they report overlapped with those detected in our study. However, the study by Johnsson et al. [15] agrees well with our findings, with overlapping significant regions on chromosomes 4, 6, 8, and 17. Their study does not specifically define the exact breed of each of the breeding lines but reported that they would have included pigs of Large White, Landrace, Duroc, Hampshire and Pietrain heritage.

### The impact of genetic differences in recombination rate in the domestic pig

The differences in average genetic map lengths and autosomal crossover rates were relatively large between some of the studied breeds, considering that they are closely related from an evolutionary perspective. Within breeds, there is genetic variation in individual crossover rates underpinned by a number of moderate effect loci. Both of these factors suggest that crossover rate as a trait has the potential to respond to selection, and in turn, could be exploited to increase the speed at which other traits of interest respond to selection [22]. However, for this to result in a significant increase in selection response for production traits, crossover rates may have to be 10 or 20 times greater, as shown by Battagin et al. [22] and it is unlikely that a breeding program would put a strong selection emphasis on recombination rates at the cost of other traits. There may also be negative impacts of increasing recombination rates that may outweigh the benefits. In human studies, mutation rates are higher at crossover sites [53] and high recombination rates have been associated with cancer [54]. In addition, and as previously mentioned, recombination can also break up beneficial allele combinations that were built up by selection [6]. A review of recombination rate variation across a broad selection of taxa by Ritz et al. [10] found that the magnitude of variation in recombination rates is similar across taxa and that mechanisms such as crossover interference lead to an upper limit that is universal across most species studied. This suggests that high recombination rates are not beneficial in spite of the potential for increased genetic variance, possibly due to biological consequences that remain to be fully understood.

## Conclusions

Our findings show that within breeds, crossover rates and recombination landscapes differ between the sexes, whereas between breeds, there is variation in crossover rates but patterns of recombination across the chromosomes are well conserved. We show that autosomal crossover count is heritable and explained by variants in genomic regions containing the following candidate genes: *RNF212*, *PRDM7, MEI1, MSH4* and *SYCP2*. Future studies should look at the mechanisms that underlie the substantial difference in recombination rates between sexes and at how individual rates may relate to reproductive traits. This study provides an example of the status of recombination rates in a domesticated species under strong selection, as well as how it may differ between relatively closely-related breeds, contributing to the understanding of variation in recombination rate in mammals in general.

## Supplementary Information

**Additional file 1: Figure S1**. Distribution of crossover counts per gamete before filtering (LR). The distribution of crossover count per gamete plotted for the LR breed as an example. The x-axis is the autosomal crossover count for a gamete and the y-axis is the count of how many gametes had a particular crossover count.

**Additional file 2: Table S1**. Effect of inbreeding on ACC. NFID are the total number of unique FID, mean F is the mean inbreeding coefficient, and E_BLUE F is the estimated effect of inbreeding with standard errors in parenthesis.

## Data Availability

The data that support the findings of this study are available from Norsvin and Topigs Norsvin but restrictions apply to the availability of these data, which were used under license for the current study, and thus are not publicly available. However, data are available from the authors upon reasonable request and with permission of Norsvin and Topigs Norsvin.
