# Supplementary figures and images for: Recombination rates in pigs differ between breeds, sexes and individuals, and are associated with the RNF212, SYCP2, PRDM7, MEI1 and MSH4 loci
Source: Genet Sel Evol. 2022 May 20;54:33. doi: 10.1186/s12711-022-00723-9 (PMC9123673; doi:10.1186/s12711-022-00723-9)

**Distribution of crossover counts per gamet**

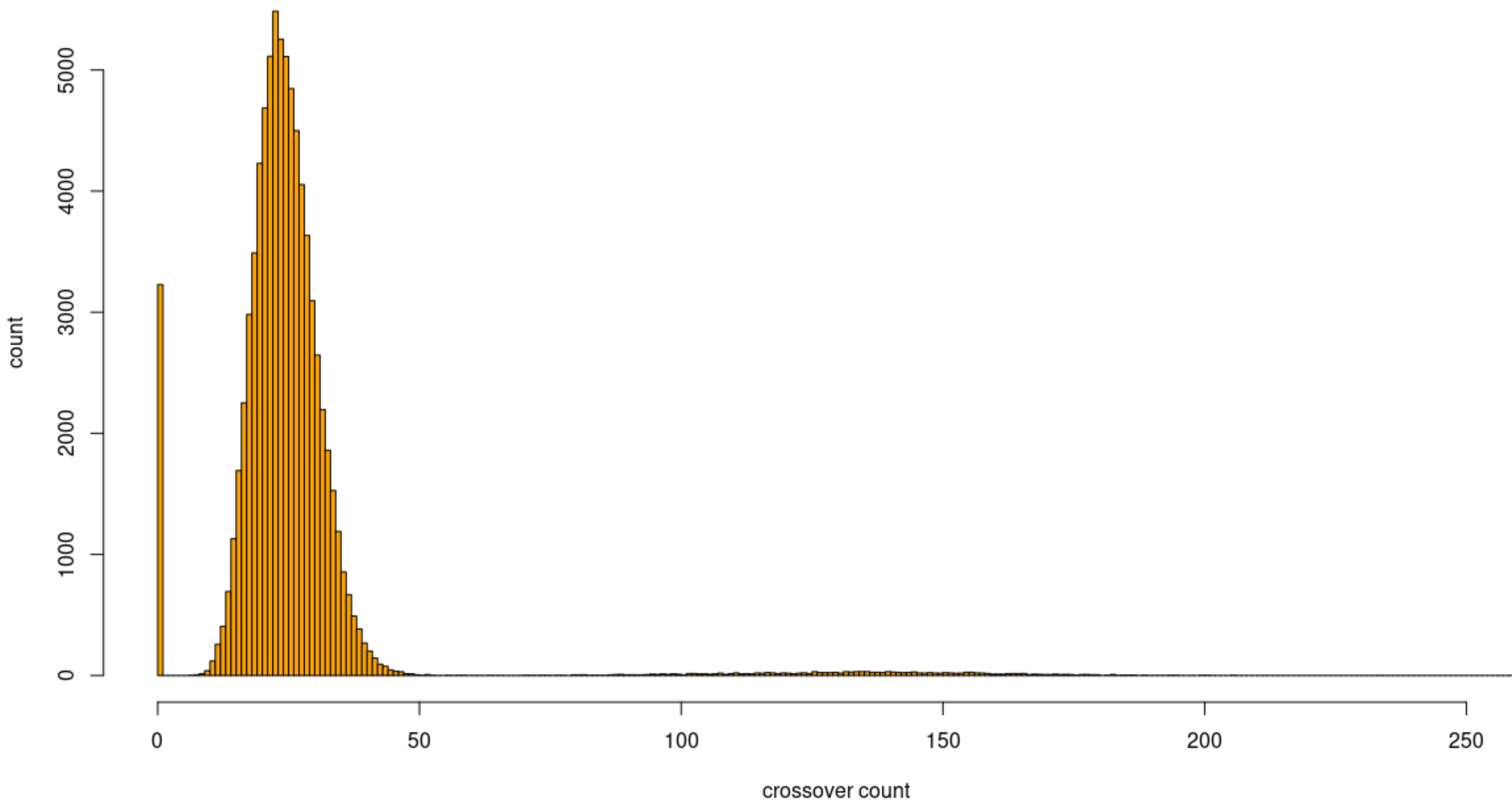

Supplement: Supplementary file 1 — Additional file 1: Figure S1. Distribution of crossover counts per gamete before filtering (LR). The distribution of crossover count per gamete plotted for the LR breed as an example. The x-axis is the autosomal crossover count for a gamete and the y-axis is the count of how many gametes had a particular crossover count. [file 12711_2022_723_MOESM1_ESM.pdf]
