# Supplementary material for: Recombination rates in pigs differ between breeds, sexes and individuals, and are associated with the RNF212, SYCP2, PRDM7, MEI1 and MSH4 loci
Source: Genet Sel Evol. 2022 May 20;54:33. doi: 10.1186/s12711-022-00723-9 (PMC9123673; doi:10.1186/s12711-022-00723-9)
Supplement: Supplementary file 2 — Additional file 2: Table S1. Effect of inbreeding on ACC. NFID are the total number of unique FID, mean F is the mean inbreeding coefficient, and E_BLUE F is the estimated effect of inbreeding with standard errors in parenthesis. [file 12711_2022_723_MOESM2_ESM.docx]

**Table S2 Effect of inbreeding on ACC**

| **Line** | **Sex** | **N FIDs** | **mean F** | **E_BLUE F (SE)** |
| --- | --- | --- | --- | --- |
| LR | Female | 4808 | 0.01 | -2.48 (0.66) |
| LR | Male | 319 | 0.04 | 2.65 (4.47) |
| LW | Female | 4695 | 0.29 | -5.53 (1.05) |
| LW | Male | 273 | 0.31 | -3-34 (1.88) |
| DU | Female | 1687 | 0.05 | -3.41 (0.95) |
| DU | Male | 192 | 0.07 | -1.87 (1.02) |
| SY | Female | 2633 | -0.03 | -3.56 (0.83) |
| SY | Male | 224 | 0.03 | -0.64 (1.12) |
| PI | Female | 1353 | -0.04 | -4.54 (1.18) |
| PI | Male | 196 | 0.01 | -3.86 (1.33) |

N_FIDs_ are the total number of unique FIDs, mean F is the mean inbreeding coefficient, and E_BLUE F is the estimated effect of inbreeding with standard errors in parenthesis.
